# Molecular Mechanisms of circRNA–miRNA–mRNA Interactions in the Regulation of Goose Liver Development

**DOI:** 10.3390/ani14060839

**Published:** 2024-03-08

**Authors:** Shuibing Liu, Chuan Li, Xiaolong Hu, Huirong Mao, Sanfeng Liu, Biao Chen

**Affiliations:** 1 College of Animal Science and Technology, Jiangxi Agricultural University, Nanchang 330045, China; 18879138275@163.com (S.L.); lichuan0122@126.com (C.L.); hulong1232007@126.com (X.H.); huirongm82@126.com (H.M.); 2 Poultry Institute, Jiangxi Agricultural University, Nanchang 330045, China; 3 Guangdong Provincial Key Laboratory of Animal Molecular Design and Precise Breeding, School of Life Science and Engineering, Foshan University, Foshan 528225, China

**Keywords:** goose liver, RNA-seq, ceRNA regulatory networks, circRNA, miRNA

## Abstract

**Simple Summary:**

This research delves into the intricate mechanisms that regulate the development of goose liver, with a particular emphasis on circular RNAs (circRNAs) and microRNAs (miRNAs). This study employed RNA sequencing (RNA-seq) to examine circRNAs and miRNAs in the liver of Sichuan white geese at three distinct stages of development. The findings revealed 11,079 circRNAs and 994 miRNAs, with notable enrichment in pathways associated with fatty acid biosynthesis and the FoxO signaling pathway among the differentially expressed circRNAs and miRNAs. Furthermore, regulatory networks involving circRNA–miRNA–mRNA interactions were constructed, and key regulatory elements such as circRNA3953 (CCNY) and circRNA1112 (TMEM106B), along with the miRNAs gga-miR-27b-3p, gga-miR-29a-3p, and gga-miR-16c-5p, were identified. These discoveries offer valuable insights into the complex interplay of circRNA–miRNA–mRNA interactions during goose liver development.

**Abstract:**

The liver, a crucial metabolic organ in animals, is responsible for the synthesis, breakdown, and transport of lipids. However, the regulatory mechanisms involving both coding and noncoding RNAs that oversee the development of the goose liver remain elusive. This study aimed to fill this knowledge gap by conducting RNA-seq to profile the expression of circular RNAs (circRNAs) and microRNAs (miRNAs) during goose liver development. We analyzed circRNAs in liver samples from Sichuan white geese at three developmental stages: posthatching day 0, 10 weeks (fast growth stage), and 30 weeks (sexual maturation stage). Our findings revealed 11,079 circRNAs and 994 miRNAs, among which the differentially expressed circRNAs and miRNAs were significantly enriched in pathways such as fatty acid biosynthesis, degradation, and metabolism. Further analysis of the target genes of the differentially expressed miRNAs revealed enrichment in pathways related to fatty acid biosynthesis, metabolism, PPAR signaling, DNA replication, and the cell cycle. We also established circRNA–miRNA–mRNA regulatory networks, identifying key regulatory factors and miRNAs. In conclusion, our study offers valuable insights into the complex interplay of circRNA–miRNA–mRNA interactions during goose liver development, and illuminates the molecular pathways that regulate this vital life function.

## 1. Introduction

Geese, a significant poultry species worldwide, are particularly valued for their liver, a delicacy. As the body’s largest gland, the liver is an accessory organ to the digestive system. In the anterior part of the body cavity, the liver is divided into two lobes, the right and left, which are directly connected by the midline. Liver lobules are hexagonal in shape and are made up of parenchymal cells (hepatocytes) and nonparenchymal cells. Nearly 80% of the liver’s volume is made up of hepatocytes, which perform numerous functions. The avian liver does not have the true lobular structure found in mammalian livers. Hepatocytes in the avian liver are organized in two-cell-layer thick plates surrounding the bile canaliculi [1].

The liver is a metabolic powerhouse in animals, serving as the primary site for fatty acid synthesis and playing a pivotal role in lipid synthesis, degradation, and transport [2]. In mature animals, the liver maintains bile acid synthesis, nutrient homeostasis, hormone production, detoxification, and immune function [3,4]. Additionally, during embryonic development and the newborn stage, the liver is a vital hematopoietic organ that produces blood cells [5]. Fatty acid synthesis in mammals primarily occurs in the liver and adipose tissue, which are also the main sites for the production of fatty acids, triacylglycerol, and phospholipids [6]. Notably, the goose liver has robust fat deposition ability and exhibits a unique fat deposition mechanism compared to that of mammals and some landfowl [7,8]. Goose fatty liver disease, which is associated with decreased inflammation and immune reactions, has shown potential for recovery, making it a valuable model for studying fatty liver in humans and other animals [9]. While there is an initial understanding of the molecular mechanisms underlying goose fatty liver disease [10], most liver functions are not fully mature during the embryonic and neonatal stages. As individuals grow, the liver undergoes numerous changes, leading to variations in liver function throughout development [11]. However, our understanding of the regulatory role of circular RNAs (circRNAs) and microRNAs (miRNAs) in goose liver development is limited.

Recent advancements in bioinformatics and sequencing technology have led to a surge in the identification of circRNAs, a type of noncoding RNA (ncRNA) characterized by covalently bonded closed loops. circRNAs are prevalent in transcripts across various species and tissues, and they have significant regulatory implications for gene expression [12,13]. Unlike linear RNAs, circRNAs exhibit greater structural stability and conservation. They serve various functions, including acting as molecular sponges for miRNAs, modulating linear RNA expression, binding to proteins, encoding proteins, and giving rise to pseudogenes, implicating them in a wide array of biological processes [14,15,16,17]. For example, circRNAs have been found to play roles in fat deposition and metabolism processes in chicken liver [18] and to contribute to liver lipid metabolism, transport, and deposition in pig liver [19].

miRNAs have a wide range of regulatory effects on gene expression [20]. By binding to the 3′ untranslated region (3′UTR) of target mRNAs, they complementarily pair with them, leading to the inhibition of mRNA translation, thus regulating gene expression posttranscriptionally [21]. MiRNAs have been identified as key players in pigeon liver development [22]. Moreover, miR-27b-5p has been reported to bind directly to insulin receptor substrate 2 (IRS2), inhibiting the PI3K/AKT signaling pathway and causing hepatic steatosis, oxidative stress, inflammation, and cell apoptosis in chickens [23].

Although there are numerous reports on goose liver, most of these studies have focused on fatty liver [24,25,26], with few reports on the different developmental stages of goose liver. This study used RNA-seq technology to systematically identify the expression profiles of circRNAs and miRNAs in goose liver at three developmental stages (posthatching day 0, 10 weeks old, and 30 weeks old). By analyzing the data, we identified circRNAs and miRNAs that were differentially expressed across these stages and predicted their functions. This result is a significant contribution to the database of goose liver circRNAs and miRNAs, providing a molecular basis for subsequent research on goose liver and a more comprehensive understanding of the biological pathways that govern goose liver development.

## 2. Materials and Methods

### 2.1. Ethics Statement

This study adhered to the ethical standards set by the Ethics Committee of Jiangxi Agricultural University (JXAULL-2017002). All geese involved were humanely euthanized.

### 2.2. Experimental Animals and Sampling

A total of nine male Sichuan white geese (*Anser cygnoides*) at three developmental stages were used in this experiment, all originating from the same group of fertilized eggs sourced from the Sichuan Agricultural University Waterfowls Breeding Farm (Ya’an, Sichuan, China). Eggs from the same batch of Sichuan white geese were also incubated and subsequently reared under identical conditions, albeit for objectives not associated with this particular study. The fertilized eggs were incubated at 37.8 °C in a fully automatic egg incubator (JT35, Jitan, China) with 60% relative humidity for 27 days. Then, all the eggs were transferred to a hatcher tray from the 28th day to the 31st day in the same incubator at 37.4 °C and 70% relative humidity. All eggs were candled on the 8th day and the 28th day, and dead embryos were removed from the incubator. The interval for egg turning was set at 2 h during the incubation, and the egg turning was halted after all the eggs were placed in the hatcher tray. All geese were reared in cages under standard temperature, humidity, and ventilation conditions on the farm. The geese aged 1–3 days lived in an environment with a temperature of 29–31 °C and 24 h of daylight. For days 4–21, the temperature range decreased to 21–28 °C with continuous 24 h of light. From day 22 to day 160, the geese were maintained in an environment with a temperature of 19 °C and natural lighting. Between day 160 and day 300, the geese lived at 19 °C with a gradual increase in lighting duration up to 16 h. A three-phase feeding system was used: a starter ration (from 0 to 8 weeks old) with 19.5% crude protein and 11.92 MJ/kg metabolizable energy, a second phase (from 8 to 25 weeks old) with 15.0% crude protein and 11.30 MJ/kg metabolizable energy, and the last phase (after 25 weeks old) with 18.0% crude protein and 11.72 MJ/kg metabolizable energy. All geese were given food and water ad libitum. The hatching, feeding, and management conditions followed strict Sichuan Agricultural University Waterfowls Breeding Farm guidelines. On the day of harvest, the geese were killed by manual exsanguination, and their sex was determined based on their anatomical characteristics. Then, the liver tissues were immediately frozen in liquid nitrogen and stored at −80 °C. Liver samples were collected from nine male geese at three developmental stages: posthatching day 0 (P group), 10 weeks old (fast growth stage, F group), and 30 weeks old (sexual maturation stage, S group). At each of these stages, liver samples were obtained from three individual geese.

### 2.3. RNA Extraction and Sequencing

Total RNA was extracted from the liver samples obtained from nine geese using TRIzol solution (Invitrogen, Carlsbad, CA, USA) according to the manufacturer’s instructions. The amount and quality of the RNA were measured using an ND-1000 spectrophotometer (NanoDrop, Wilmington, DE, USA). The integrity of the RNA was confirmed by analyzing it on an Agilent 2100 bioanalyzer (Agilent Technologies, Palo Alto, CA, USA). A total of 5 µg of RNA was loaded to deplete rRNA with a Ribo-Zero^TM^ rRNA Removal Kit (Illumina, San Diego, CA, USA). Noncircular RNAs were degraded by ribonuclease R (Epicentre, Madison, WI, USA). The cleaved circRNAs, which were enriched in the sample, were subsequently utilized for cDNA synthesis. The blunt termini of the cDNA strands were processed with an A-base to facilitate their connection to the barcoded adapters. Following the established Illumina standard library preparation protocol, the library was constructed and sequenced using an Illumina NovaSeq^TM^ 6000 platform (LC Bio, Hangzhou, Zhejiang, China) according to the manufacturer’s instructions. For the detailed steps of circRNA sequencing, please refer to our previous paper [27].

Libraries for small RNA sequencing were created following the manufacturer’s instructions using TruSeq Small RNA Sample Prep Kits (Illumina, San Diego, CA, USA). In brief, whole RNA was ligated using 3′ and 5′adaptor reads. This was followed by the amplification of RNA using adaptors via RT–PCR and subsequent gel-based filtration. According to the vendor’s recommendations, the final products were loaded onto a HiSeq 2500 for sequencing (LC Bio, Hangzhou, Zhejiang, China). Our previous paper provides detailed miRNA sequencing methods and steps [20].

### 2.4. Sequencing Data Analysis

For circRNA sequencing, a comprehensive description of the analysis procedures can be found in our previous publication [27]. Briefly, following a series of filtering, quality control, and genome alignment (https://www.ncbi.nlm.nih.gov/genome/?term=Anser+cygnoides, accessed on 18 December 2023) steps utilizing Cutadapt [28], FastQC (https://www.bioinformatics.babraham.ac.uk/projects/fastqc/, accessed on 16 December 2023), Bowtie2 [29], Tophat2 [30], Tophat-fusion [31], CIRCExplorer2 [32,33], CIRI [34], and circular RNA identification processes, the presence of circular RNAs in goose liver was successfully determined. The circRNA expression levels are indicated using spliced reads per billion mapped reads (srpbm). For comparisons, edge R was utilized, with a |log2 fold change| ≥ 1 and a *p*-value < 0.05 indicating differential expression. The raw data and processing files for all sequencing experiments are available from the China National Center for Bioinformation, accessed on 9 January 2024, with the accession number CRA014346.

For the miRNA sequencing, please refer to our previous paper for the naming, characterization, and analysis of miRNAs [20]. To clarify the relationship between miRNAs in the sequencing data and the reported miRNAs, we used a unique miRNA nomenclature. The following conventions were used: when a miRNA matched with the miRBase database, L-n and R-n represented a loss of n-bases at the left and right ends of the reported miRNA, respectively. Conversely, L + n and R + n indicate the addition of n bases at the left and right ends of the reported miRNA, respectively. Additionally, 2ss5TC13TA indicated that the fifth base T was substituted with C (where ss indicates substitution), and the 13th base T was substituted with A (the initial 2 indicates a total of two bases replaced). Furthermore, newly identified miRNAs were denoted by “PC” (predicted candidate), and the positions of the 5p or 3p arms were marked. The relative expression of miRNAs was calibrated, and the differential expression of miRNAs was determined using the criteria of a |log2 fold change| ≥ 1 and *p* < 0.05. We conducted enrichment analyses of all paternal genes of circRNAs and target genes of miRNAs using the edge R package (version 3.22.5).

### 2.5. Construction of the RNA Regulation Network

The competing endogenous RNA (ceRNA) network, which links circRNAs, miRNAs, and mRNAs, was constructed to study interactions among differentially expressed circRNAs (DEcircRNAs), differentially expressed miRNAs (DEmiRNAs), and differentially expressed mRNAs (DEmRNAs). It is important to note that the DEmRNA data were derived from unpublished sequencing data from the same sample (China National Center for Bioinformation, GSA: CRA012842). Based on TargetScan v5.0 [35] (with a score threshold of 50) and Miranda v3.3a [36] (with an energy threshold of −10), interactions between circRNA and mRNA were studied, with strict score and energy thresholds to ensure the selection of highly reliable interactions with likely biological significance.

Initially, we undertook a broad analysis of interactions and then refined our criteria to focus on the most consequential interactions. For the F vs. P group, we set stringent thresholds: the minimum TargetScan_score for miRNA–mRNA interactions was 95 and the maximum Miranda_Energy was −90, the minimum TargetScan_score for miRNA–circRNA interactions was 70, and the maximum Miranda_Energy was −20. When examining the S vs. F group, we adjusted our criteria slightly. The minimum TargetScan_score for miRNA–mRNA interactions was 90, the maximum Miranda_Energy was −50, the minimum TargetScan_score for miRNA–circRNA interactions was 50, and the maximum Miranda_Energy was −10. Moving on to the S vs. P group, we raised the bar again. The minimum TargetScan_score for miRNA–mRNA interactions was 95, the maximum Miranda_Energy for miRNA–circRNA interactions was −95, the minimum TargetScan_score for miRNA–circRNA interactions was 80, and the maximum Miranda_Energy was −25. To visualize this intricate network, we utilized Cytoscape v3.8.2 [37], which greatly facilitated our understanding of the regulatory interactions at play.

## 3. Results

### 3.1. Identification and Characteristics of circRNAs during Goose Liver Development

**Figure 1:**
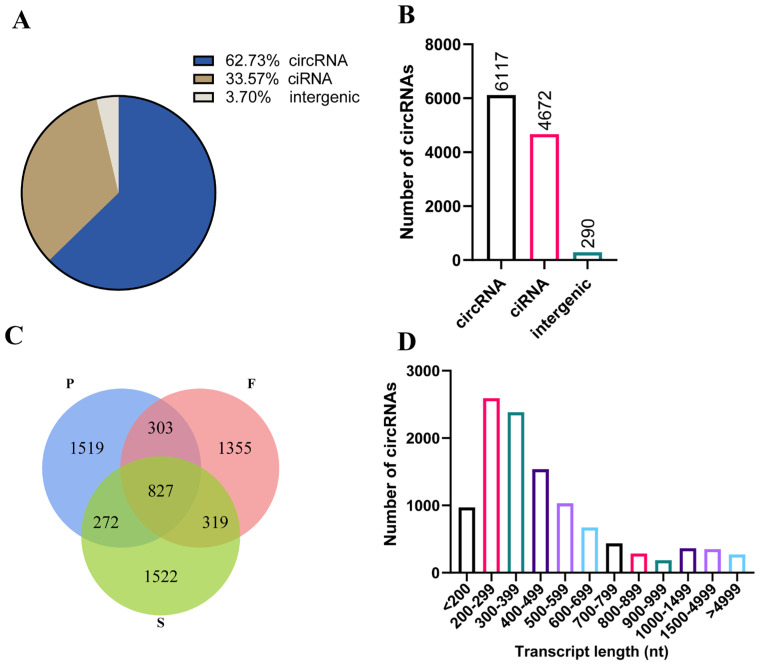
Circular RNA expression profiling. (**A**) The types and proportions of circular RNAs. circRNA: exon–derived circular RNA. ciRNA: intron–derived circular RNA. intergenic: intergenic–derived circular RNA. (**B**) The numbers of different kinds of circular RNA. (**C**) Venn diagrams indicating the overlap of circular RNAs expressed at different developmental stages. (**D**) Length distribution of circular RNA reads. nt: nucleotides.

We used the Illumina NovaSeq™ 6000 platform to sequence the liver samples from nine geese at three different stages (P group: posthatching day 0, F group: 10 weeks old, S group: 30 weeks old). We obtained a total of 783,588,382 valid reads with over 95.70% Q30, which were used to identify circRNAs (Table 1). We found that all liver samples from nine geese exhibited a sequence alignment of more than 66.14% to the reference genome of geese in the NCBI database. To identify circular RNA, we applied criteria including a mix of mismatches ≤ 2 and back-spliced junctions ≥ 1, as well as two splice sites within 100 kb of the genome. Following these standards, we generated a total of 18,906 circular RNAs from 12,962 circRNA-hosting genes out of 7,794,983 candidate back-spliced junction reads (Table 1). Of these circular RNAs, exon-derived circular RNA (circRNA) accounted for the majority (62.73%), followed by intron-derived circular RNA (ciRNA) and intergenic-derived circular RNA (intergenic). Among these, 6117 were circRNAs, 4672 were ciRNAs, and 290 originated from intergenic reads (Figure 1A,B). We found that 827 circRNAs coexisted in all groups. Additionally, 1519 circRNAs were exclusively expressed in the P group, 1355 in the F group, and 1522 in the S group (Figure 1C). The lengths of the circular RNAs were predominantly within the range of 200–500 nt (Figure 1D). In summary, the RNA sequencing data demonstrated extensive expression of circular RNAs in goose livers.

### 3.2. Identification of DEcircRNAs during Goose Liver Development

**Figure 2:**
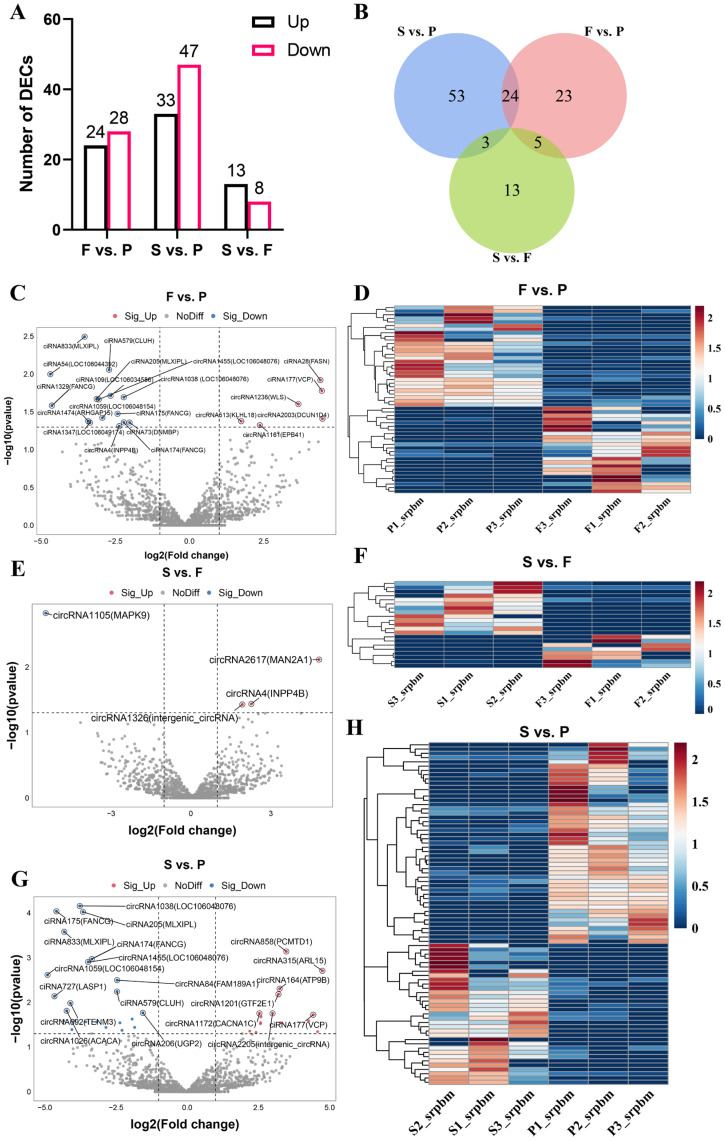
Differential expression of circular RNAs during liver development in geese. (**A**) The numbers of upregulated and downregulated DEcircRNAs in the three comparison groups (F vs. P, S vs. P, S vs. F); (**B**) Venn diagrams showing the overlap of DEcircRNAs in the three comparison groups; volcano plots indicating the expression variation of circular RNAs in F vs. P (**C**), S vs. P (**E**), and S vs. F (**G**); heatmaps showing the expression levels of DEcircRNAs in F vs. P (**D**), S vs. F (**F**), and S vs. P (**H**).

To investigate the possible roles of circRNAs detected during goose liver development, we performed a differential expression analysis by applying the following conditions: |log2 fold change| ≥ 1 and *p*-value < 0.05. We identified 52 DEcircRNAs in F vs. P, such as circRNA1474 (ARHGAP15), circRNA4 (INPP4B), circRNA2003 (DCUN1D4), and circRNA1236 (WLS). Moreover, there were 21 DEcircRNAs between the F group and the S group, including circRNA1105 (MAPK9) and circRNA2617 (MAN2A1). Additionally, there were a total of 80 DEcircRNAs between the P group and the S group, for example, circRNA206 (UGP2), circRNA692 (TENM3), circRNA315 (ARL15), and circRNA1201 (GTF2E1) (Figure 2A,C,E,G, and Supplementary Table S1). There were 24 DEcircRNAs in both F vs. P and S vs. P, 5 DEcircRNAs in both F vs. P and S vs. F, and 3 DEcircRNAs in both S vs. P and S vs. F (Figure 2B). We performed a cluster analysis for the DEcircRNAs identified in the three comparison groups, revealing significant differences in the upregulation and downregulation of circular RNAs between each comparison group (Figure 2D,F,H). This analysis revealed significant differences in the expression of circRNAs among the different groups, indicating potential regulatory roles for circRNAs during the maturation of goose liver.

### 3.3. Enrichment Analysis of DEcircRNAs during Goose Liver Development

**Figure 3:**
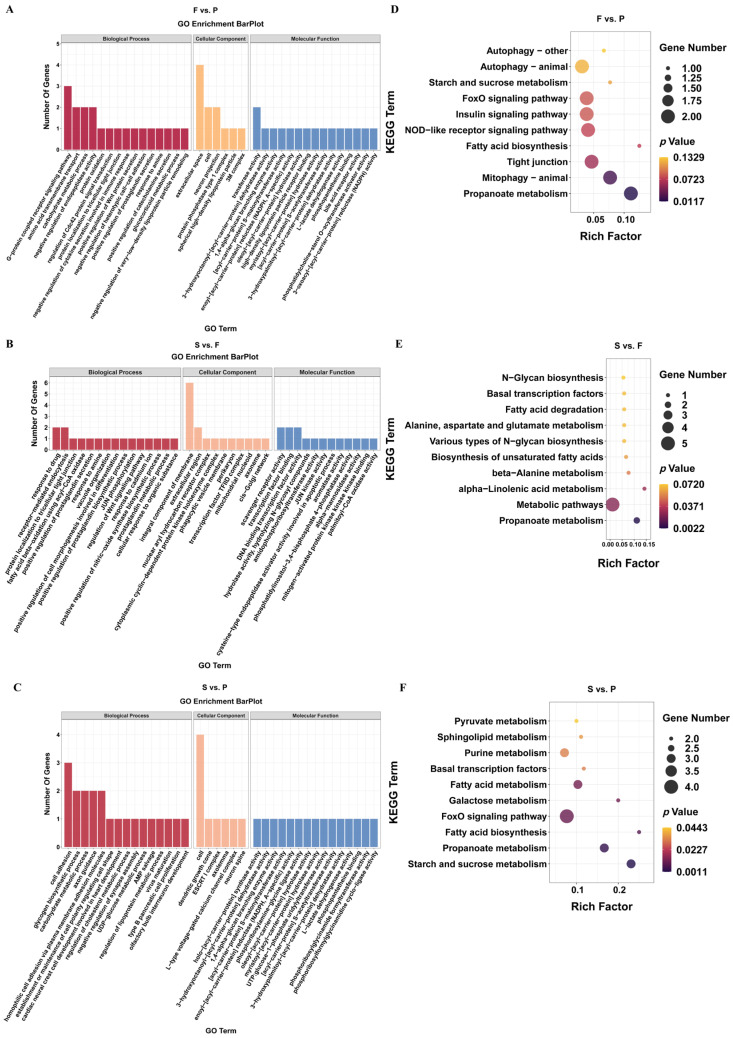
Enrichment analysis of the source genes of the DEcircRNAs during liver development in geese. The source genes of the DEcircRNAs were classified based on GO functions in F vs. P (**A**), S vs. P (**B**), and S vs. F (**C**). The 10 most enriched KEGG signaling pathways of the source genes of the DEcircRNAs in F vs. P (**D**), S vs. P (**E**), and S vs. F (**F**).

The expression of genes or transcripts can be controlled by circular RNAs through their parental genes [38]. The parental genes of the DEcircRNAs were subjected to a GO analysis to determine the role of circRNAs in the development of goose liver (Supplementary Table S2). In F vs. P, we observed a significant enrichment (*p* < 0.05) of parental genes for DEcircRNAs in 197 GO terms, including amino acid transmembrane transport, G-protein-coupled receptor signaling pathway, extracellular space, cell, and transferase activity (Figure 3A). Similarly, in S vs. F, we found significant enrichment (*p* < 0.05) of parental genes for DEcircRNAs in 108 GO terms, including response to drug, receptor-mediated endocytosis, integral component of membrane, extracellular region, scavenger receptor activity, and transcription factor binding (Figure 3B). Furthermore, in S vs. P, we observed significant enrichment (*p* < 0.05) of parental genes for DEcircRNAs in 156 GO terms, including cell adhesion, glycogen biosynthetic process, cell, dendritic growth cone, holo-[acyl-carrier-protein] synthase activity, and 3-hydroxyoctanoyl-[acyl-carrier-protein] dehydratase activity (Figure 3C). Additionally, we conducted a KEGG analysis of the parental genes associated with DEcircRNAs in the three comparisons (Supplementary Table S3). The analysis revealed significant enrichment of parental genes for DEcircRNAs in the propanoate metabolism and mitophagy–animal pathways (*p* < 0.05) in F vs. P (Figure 3D). Moreover, in S vs. F, the source genes for DEcircRNAs were significantly enriched in the propanoate metabolism, metabolic pathways, and alpha-linolenic acid metabolism pathways (*p* < 0.05) (Figure 3E). Furthermore, in S vs. P, the parental genes of the DEcircRNAs were markedly enriched in 10 pathways related to starch and sucrose metabolism, propanoate metabolism, and fatty acid biosynthesis (*p* < 0.05) (Figure 3F).

### 3.4. Expression Profiling and Differential Expression of miRNAs during Goose Liver Development

**Figure 4:**
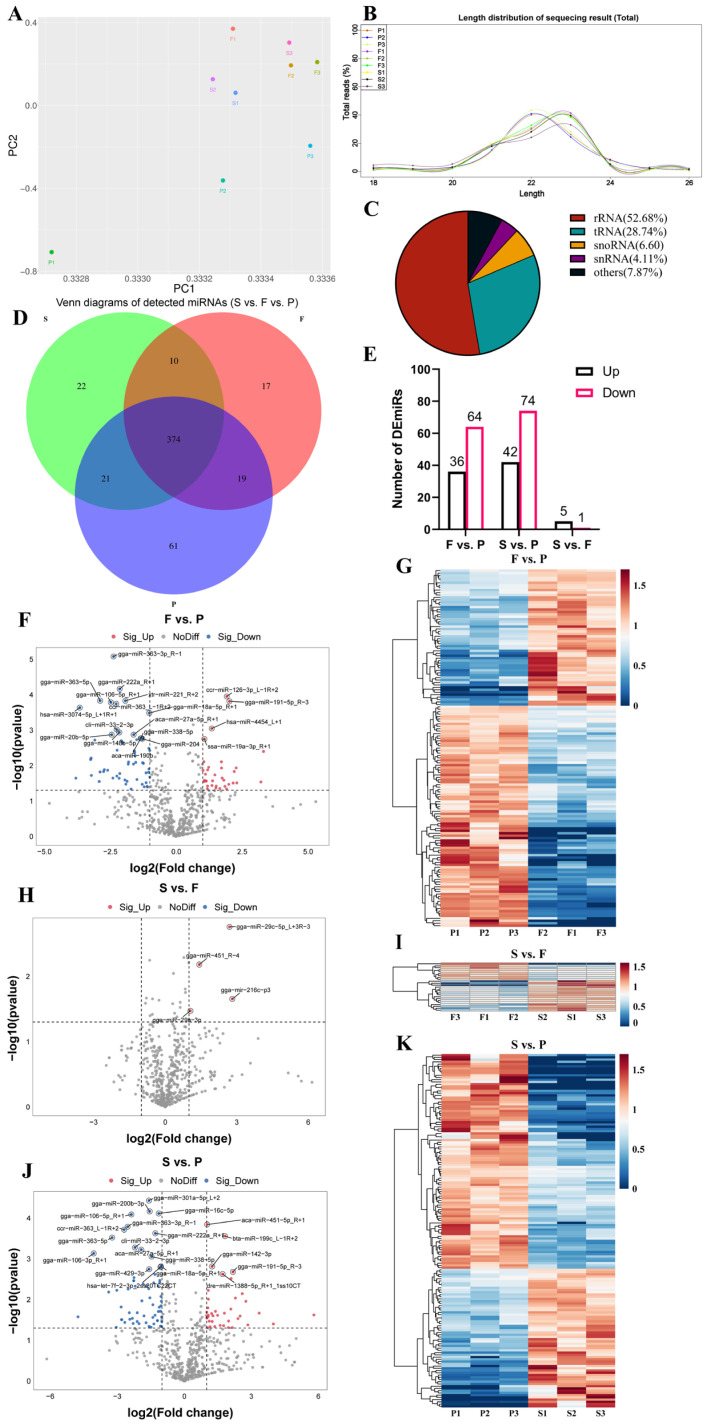
Differential expression of miRNAs during liver development in geese. (**A**) Principal component analysis (PCA) plot illustrating the expression of miRNAs across each sample; (**B**) length distribution percentage of small RNA reads during liver development of geese; (**C**) types and proportions of detected small RNAs; (**D**) Venn diagrams showing the overlap of DEmiRNAs in the three comparison groups; (**E**) numbers of upregulated and downregulated miRNAs; volcano plots indicating the expression variation of miRNAs in F vs. P (**F**), S vs. F (**H**), and S vs. P (**J**). The volcano plots were produced according to the following conditions: |log2 fold change| ≥ 1 and *p* < 0.05. Heatmaps showing the expression levels of DEmiRNAs in F vs. P (**G**), S vs. F (**I**), and S vs. P (**K**).

Using the Illumina HiSeq 2500 platform, we sequenced a total of 102,397,736 reads from nine geese representing three developmental stages of the liver. After quality control and filtering, we obtained 82,843,300 clean reads. After removing reads shorter than 18 nt and longer than 26 nt, we identified a total of 69,892,315 valid reads (Table 2). We used the principal component analysis (PCA) to analyze the samples based on gene expression data (Figure 4A). In the P group, the three geese were widely separated, while in the F and S groups, the three geese were closely related. We observed differences in miRNA expression among the three groups. The lengths of these miRNAs were concentrated in the range of 20–24 nt, consistent with the characteristic length of miRNAs, confirming the reliability of the dataset (Figure 4B). After excluding other types of small RNAs, such as rRNA and tRNA (Figure 4C), we found that 994 miRNAs were expressed in the liver samples from the nine geese (Supplementary Table S4), whereby 374 miRNAs were expressed in all three groups, 61 miRNAs were exclusively detected in the P group, 17 miRNAs were uniquely detected in the F group, and 22 miRNAs were solely detected in the S group (Figure 4D). To explore the potential functions of miRNAs expressed during goose liver development, we conducted a differential expression analysis with the conditions of a |log2 fold change| ≥ 1 and *p* < 0.05 (Supplementary Table S4). There were 100 DEmiRNAs between the F group and P group, such as gga-miR-363-5p and gga-miR-191-5p_R-3. Additionally, there were 116 DEmiRNAs between the S group and P group, including gga-miR-200b-3p and gga-miR-142-3p1. Furthermore, there were six DEmiRNAs between the S group and F group, such as gga-miR-29c-5p_L+3R-3 and gga-miR-29a-3p (Figure 4E). The volcano plots provide a visual representation of the differential expression and distribution of miRNAs (Figure 4F,H,J). Furthermore, the heatmaps illustrate the differential expression of miRNAs among the three comparison groups (Figure 4G,I,K). These results indicate the presence of DEmiRNAs during goose liver development.

### 3.5. Enrichment Analysis of the Target Genes of DEmiRNAs during Goose Liver Development

**Figure 5:**
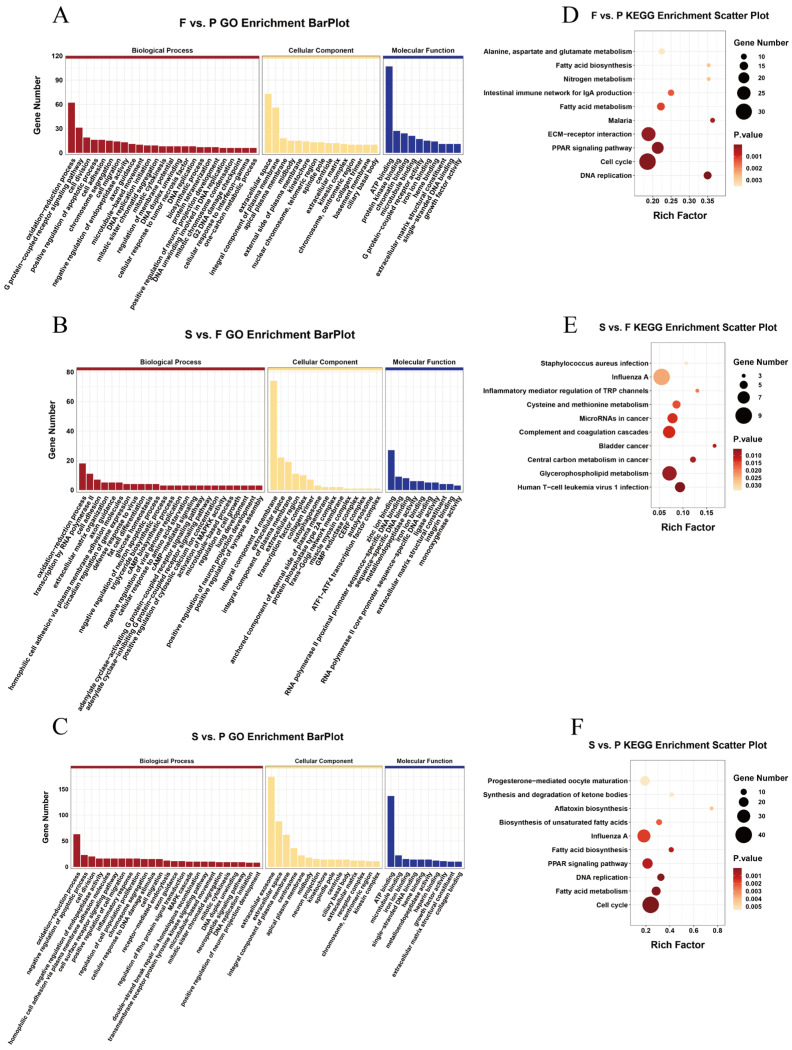
Enrichment analysis of the DEmiRNA target genes during goose liver development. The DEmiRNA target genes were enriched in F vs. P (**A**), S vs. F (**B**), and S vs. P (**C**). The top 25, 15, and 10 terms are presented. The 10 most enriched KEGG signaling pathways of the DEmiRNA target genes in F vs. P (**D**), S vs. P (**E**), and S vs. F (**F**) are shown.

A GO enrichment analysis was subsequently conducted on the target genes of the DEmiRNAs to further understand their functions (Supplementary Table S5). The GO enrichment analysis results indicated considerable enrichment of the DEmiRNA target genes in 283 GO terms in F vs. P. These terms predominantly included processes such as oxidation–reduction, G protein-coupled receptor signaling pathways, and cell division (*p* < 0.05) (Figure 5A). Similarly, in the S vs. F, the DEmiRNA target genes were notably enriched in 310 GO terms, focusing on processes such as oxidation–reduction, transcription by RNA polymerase II, and cell adhesion (*p* < 0.05) (Figure 5B). Furthermore, in the S vs. P, the DEmiRNA target genes were found to be significantly enriched in 331 GO terms, emphasizing processes such as oxidation–reduction, negative regulation of apoptotic processes, and cell division (*p* < 0.05) (Figure 5C). The differential expression of miRNAs in the three comparison groups was also subjected to a KEGG enrichment analysis (Supplementary Table S6). Significant enrichment (*p* < 0.05) of the DEmiRNA target genes was observed in 29 KEGG pathways, including DNA replication, the cell cycle, and the PPAR signaling pathway, in F vs. P (Figure 5D). Similarly, in S vs. F, the target genes of the DEmiRNAs were markedly enriched (*p* < 0.05) in 16 KEGG pathways, such as glycerophospholipid metabolism, human T-cell leukemia virus 1 infection, and central carbon metabolism in cancer (Figure 5E). Moreover, S vs. P showed significant enrichment (*p* < 0.05) of DEmiRNA target genes in 29 KEGG pathways, including the cell cycle, fatty acid metabolism, and DNA replication (Figure 5F).

### 3.6. Construction of the ceRNA Interaction Regulatory Network

**Figure 6:**
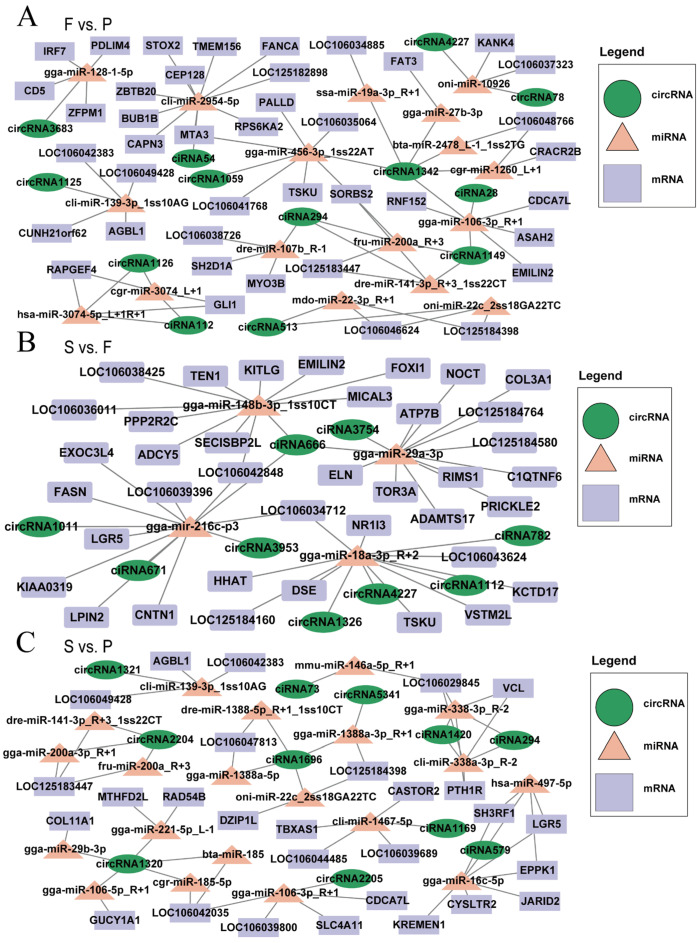
circRNA–miRNA–mRNA ceRNA regulatory network. The potential ceRNA regulatory networks involving circRNA–miRNA–mRNA interactions in F vs. P (**A**), S vs. F (**B**), and S vs. P (**C**). The purple rectangles denote mRNAs, the peach triangles represent miRNAs, and the green oval shapes represent circRNAs.

Research has suggested that circRNAs serve as miRNA sponges and that miRNAs regulate mRNA functions [39]; therefore, we established a circRNA–miRNA–mRNA interaction regulatory network (Supplementary Table S7). This network consolidates the interactive connections between DEmiRNAs and their targets within the realm of DEcircRNAs. The results revealed the construction of circRNA–miRNA–mRNA regulatory networks in three different comparison groups: F vs. P, S vs. F, and S vs. P. In F vs. P, networks were built based on specific criteria for miRNA–mRNA (TargetScan_score ≥ 95, Miranda_Energy ≤ −90) and miRNA–circRNA (TargetScan_score ≥ 70, Miranda_Energy ≤ −20), resulting in a network consisting of 41 mRNAs, 13 circRNAs, and 17 miRNAs (Figure 6A). In S vs. F, networks were established using other criteria for miRNA–mRNA (TargetScan_score ≥ 90, Miranda_Energy ≤ −50) and miRNA–circRNA (TargetScan_score ≥ 50, Miranda_Energy ≤ −10), yielding a network comprising 38 mRNAs, 9 circRNAs, and 4 miRNAs (Figure 6B). Finally, in the S vs. P, networks were also structured with different miRNA–mRNA (TargetScan_score ≥ 95, Miranda_Energy ≤ −95) and miRNA–circRNA (TargetScan_score ≥ 80, Miranda_Energy ≤ −25) criteria, resulting in a network containing 28 mRNAs, 11 circRNAs, and 20 miRNAs (Figure 6C). Key regulatory factors were identified, including circRNA1149 (PPP1R15B), circRNA1126 (LOC106049357), circRNA3953 (CCNY), circRNA1112 (TMEM106B), gga-miR-27b-3p, gga-miR-29a-3p, gga-miR-29b-3p, and gga-miR-16c-5p. These complex circRNA–miRNA–mRNA regulatory networks pave the way for deeper exploration into how these genes and ncRNAs influence goose liver development.

## 4. Discussion

The goose is one of the most economically valuable poultry species, providing meat, eggs, and highly prized liver. Sichuan white geese are renowned for their superior breeding performance in China [40]. The liver is a complex organ that develops through interactions among multiple cell types and tissues and is regulated by various transcription factors, epigenetic modulators, and noncoding RNAs [41]. Increasing evidence suggests that liver development is tightly and dynamically regulated by a variety of circRNAs and miRNAs [19,42]. The aim of this study was to compare the expression of circRNAs and miRNAs in the livers of Sichuan white geese at three different developmental stages using high-throughput sequencing. We profiled the expression of circRNAs and miRNAs in liver samples from nine geese at posthatching day 0 (P group), 10 weeks old (fast growth stage, F group), and 30 weeks old (sexual maturation stage, S group) and identified and analyzed DEcircRNAs and their parental genes to elucidate their molecular functions. We also examined DEmiRNAs and their target genes to provide a comprehensive understanding of the regulatory mechanisms underlying goose liver development.

circRNAs are endogenous noncoding RNAs that are found in various species and tissues and play important roles in animal tissues [43]. Previous research has shown that circIPO11 can activate the hedgehog signaling pathway to drive the self-renewal of liver cancer-initiating cells and promote the proliferation of hepatocellular carcinoma (HCC) cells [44]. In this study, we identified 6448, 6033, and 6442 circRNAs in goose liver at different developmental stages, suggesting that circRNAs play important roles in goose liver development. Most of the circRNAs were derived from exons, which is in agreement with studies in chicken and duck muscles but different from studies in duck follicles, suggesting tissue-specific reverse splicing [27,45,46]. We also detected 52 (F vs. P), 21 (S vs. F), and 80 (S vs. P) DEcircRNAs at the F, S, and P stages, respectively.

There was a significant upregulation and enrichment in the biological processes of Wnt protein secretion in the F vs. P group of circRNA1236 (WLS). The Wnt ligand secretion mediator (WLS) gene encodes a protein involved in the Wnt signaling pathway that regulates the sorting and secretion of Wnt proteins through a feedback mechanism [47]. The WLS protein also affects the expression, subcellular localization, binding, and organelle-specific binding of Wnt proteins [48]. The Wnt signaling pathway plays a key role in regulating liver size and metabolic function in adult individuals. Studies have shown that eliminating *WLS* in mice causes a significant decrease in liver mass, accompanied by changes in liver metabolic zoning and lipid metabolism. Moreover, the knockout of the *WLS* gene inhibits the growth of most liver cancer cells [49,50].

In S vs. F, circRNA1105 (MAPK9) was significantly downregulated. The mitogen-activated protein kinase 9 (MAPK9) gene encodes a protein that belongs to the MAP kinase family [51]. Biochemical signals are integrated by MAP kinases, which play a role in cell proliferation, differentiation, transcriptional regulation, and development [52,53]. In a partial hepatectomy mouse model, it was shown that MAPK9 may play a role in hepatocyte proliferation by inactivating p38-MAPK during DNA replication [54]. In this study, circRNA1105 (MAPK9) was enriched in signaling pathways related to GnRH signaling, Wnt signaling, FoxO signaling, and MAPK signaling, suggesting its significant role in liver development.

In the S vs. P group, circRNA206 (UGP2) was significantly downregulated. The UDP-glucose pyrophosphorylase 2 (UGP2) gene encodes an enzyme that plays a vital role in the interconversion of mammalian carbohydrates, converting glucose-1-phosphate to MgUTP and forming UDP-glucose and MgPPi [55]. In liver cancer cells, overexpression of *UGP2* can promote cell migration and invasion while enhancing extracellular glycogen production [56]. These results indicate that circRNA1236 (WLS), circRNA1105 (MAPK9), and circRNA206 (UGP2) may play important roles in liver development, and further investigations are needed to elucidate the impact of these circRNAs on liver development.

miRNAs are known to regulate gene expression by directly targeting mRNAs and interacting with mRNAs and noncoding RNAs through ceRNA regulation [57]. miR-122 is a highly conserved liver-specific miRNA in vertebrates that is essential for maintaining liver homeostasis [58]. Its significance is evident from its role in promoting hepatic steatosis by targeting genes involved in lipid metabolism, such as fructose-bisphosphate B (ALDOB) [59]. Moreover, miR-122 has been shown to bind to circPI4KB and transport it to extra-hepatocytes, thus decreasing the protective role of miR-122 in targeting mRNA and preventing lipid deposition [60]. In this study, we identified several DEmiRNAs, such as gga-miR-27b-3p, gga-miR-29a-3p, and gga-miR-29b-3p. Notably, circRanGAP1 functions as a target of miR-27b-3p and may promote the onset of hepatocellular carcinoma (HCC) through the miR-27b-3p/NRAS/ERK axis [61]. Furthermore, the lncRNA MSTRG4710 promotes preadipocyte proliferation and differentiation through miR-29b-3p/IGF1 [62]. Interestingly, the levels of miR-29a-3p and miR-29b-3p, which are members of the miR-29 family, have been demonstrated to increase in various tissue and cell models of metabolic diseases i.e., obesity, insulin resistance, and type 2 diabetes [63]. Hepatic insulin resistance can be improved by either eliminating or depleting miR-29 in models of insulin disarray and type 2 diabetes [64,65,66]. We identified key regulatory factors such as gga-miR-27b-3p, gga-miR-29a-3p, gga-miR-29b-3p, and gga-miR-16c-5p. These ceRNA regulatory networks are expected to facilitate further exploration of the molecular mechanisms underlying goose liver development in the future.

The liver plays a crucial role in storing and converting nutrients for energy. It stores excess glucose as glycogen and converts it into fat [67]. When there is a shortage of nutrients, the liver breaks glycogen down into glucose or increases gluconeogenesis to maintain blood glucose levels [68,69,70]. Geese have a unique lipid metabolism pattern in which the liver, not adipose tissue, is the main organ for lipid storage [7]. In our study, we detected significantly greater expression levels of circRNA971 (MAPK1) and circRNA754 (SFMBT2) in the posthatching day 0 group than in the 30-week-old sexual maturation group. Notably, the parental genes of circRNA971 and circRNA754, Mitogen-Activated Protein Kinase 1 (MAPK1) and Scm-Like With Four Mbt Domains 2 (SFMBT2), respectively, are implicated in the control of cell proliferation, highlighting their crucial role in this biological process [71,72,73]. Our findings revealed that circRNAs obtained during the three developmental stages of goose liver development were enriched mainly in parental genes involved in metabolic pathways such as fatty acid biosynthesis, unsaturated fatty acid biosynthesis, fatty acid metabolism, and the FoxO signaling pathway. Similarly, the DEmiRNA target genes were predominantly enriched in pathways such as fatty acid biosynthesis, fatty acid metabolism, glycerophospholipid metabolism, the PPAR signaling pathway, DNA replication, and the cell cycle. DNA replication and cell cycle pathways are crucial for liver development and hepatocyte proliferation [74,75]. In overfed geese with fatty liver, genes related to fatty acid metabolism, PPAR signaling, and cell cycle pathways are significantly differentially expressed [76]. Studies have suggested that the PPAR pathway may play an essential role in lipid metabolism in goose liver [25]. Forkhead O transcription factors (FOXOs) are key regulators of glucose and lipid balance in the liver and are directly influenced by insulin signaling [77]. Overall, our results suggest a potential link between the liver at the P stage and the cell cycle and liver growth. Moreover, the liver during the F and S stages appears to be more closely associated with fat metabolism.

## 5. Conclusions

Overall, we explored the expression patterns of circRNAs and miRNAs in goose liver at three developmental stages. We identified 52 DEcircRNAs and 100 DEmiRNAs between the F group and the P group, 21 DEcircRNAs and 6 DEmiRNAs between the F group and the S group, and 80 DEcircRNAs and 116 DEmiRNAs between the P group and the S group. Moreover, we identified potential core circRNAs and miRNAs among these DEcircRNAs and DEmiRNAs, including circRNA1236 (WLS), circRNA1105 (MAPK9), circRNA206 (UGP2), circRNA1149 (PPP1R15B), circRNA1126 (LOC106049357), circRNA3953 (CCNY), circRNA1112 (TMEM106B), miR-27b-3p, miR-29a-3p, miR-29b-3p, and miR-16c-5p. We also revealed potential ceRNA regulatory networks involving circRNA–miRNA–mRNA interactions, which provide more insight into the roles of circRNAs and miRNAs in modulating goose liver development. These results pave the way for further research on goose liver development.

## Figures and Tables

**Table 1:** Sequencing data generated from the RNase R digested library.

Identification Number	Read	Q30%	Valid Reads	Mapped Reads	Back-Spliced Junctions Reads	Number of circRNA-Hosting Genes	Number of circRNAs
P1	182,770,542	96.02	94,846,238	63,808,104 (67.28%)	920,007 (0.97%)	1573	2385
P2	194,923,376	95.70	89,396,850	59,130,157 (66.14%)	855,279 (0.96%)	1356	1943
P3	167,873,330	96.29	96,477,610	69,367,040 (71.90%)	844,581 (0.88%)	1458	2120
F1	130,003,024	96.80	81,026,888	60,809,218 (75.05%)	813,446 (1.00%)	1486	2176
F2	134,426,502	96.67	91,167,744	67,387,484 (73.92%)	1,268,977 (1.39%)	1315	1882
F3	151,546,164	96.65	86,646,924	64,557,846 (74.51%)	830,104 (0.96%)	1351	1975
S1	151,401,712	96.35	88,253,086	63,009,145 (71.40%)	843,406 (0.96%)	1497	2180
S2	149,935,366	96.66	88,416,132	64,772,234 (73.26%)	782,180 (0.88%)	1689	2529
S3	127,097,270	96.75	67,356,910	51,322,862 (76.20%)	637,003 (0.95%)	1237	1733

**Table 2:** Summary of the miRNA sequencing data.

Identification Number	Raw Reads	Clean Reads	Clean Ratio, %	Valid Reads	Valid Ratio, %
P1	10,696,086	8,876,088	82.98	7,316,506	68.40
P2	11,729,357	9,302,214	79.31	7,922,998	67.55
P3	11,681,199	9,556,620	81.82	8,077,834	69.15
F1	10,880,983	9,258,042	85.08	7,994,024	73.47
F2	12,183,931	10,423,708	85.55	8,759,628	71.89
F3	10,290,009	8,546,903	83.06	7,324,075	71.18
S1	11,750,317	9,597,940	81.69	7,928,524	67.47
S2	11,457,809	9,158,427	79.93	7,615,894	66.47
S3	11,728,045	8,123,358	69.27	6,952,832	59.28

## Data Availability

The raw data and processing files for all sequencing experiments are available at the China National Center for Bioinformation, with the accession numbers CRA014346 and CRA012842. The data were last accessed on 9 January 2024.

## Supplementary Materials

The supplementary files can be downloaded at: https://www.mdpi.com/article/10.3390/ani14060839/s1, Supplementary Table S1: Summary of circRNAs during goose liver development. Supplementary Table S2: List of the GO analysis of the source genes of DEcircRNAs during goose liver development. Supplementary Table S3: List of the KEGG analysis of DEcircRNA source genes during goose liver development. Supplementary Table S4: Summary of miRNA expression during goose liver development. Supplementary Table S5: List of the GO analysis of DEmiRNA target genes during goose liver development. Supplementary Table S6: List of the KEGG analysis of DEmiRNA target genes during goose liver development. Supplementary Table S7: List of ceRNA regulatory networks during goose liver development.
